# Caffeine enhances performance regardless of fueling strategy; however, high carbohydrate availability is associated with improved training speeds compared with ketogenic diet

**DOI:** 10.1017/S0007114525105084

**Published:** 2025-10-14

**Authors:** Louise M. Burke, Lucy Merrell, Ida A. Heikura, Rita Civil, Avish P. Sharma, Jill J. Leckey, Alannah K. A. McKay

**Affiliations:** 1 Mary MacKillop Institute for Health Research, Australian Catholic University, Melbourne, VIC, Australia; 2 Centre for Nutrition, Exercise and Metabolism, University of Bath, Bath, UK; 3 School of Sport, Exercise & Rehabilitation Sciences, College of Life and Environmental Sciences, University of Birmingham, Birmingham, UK; 4 Victorian Institute of Sport, Melbourne, VIC, Australia; 5 South Australian Sports Institute, Adelaide, SA, Australia

**Keywords:** Carbohydrate, Endurance, Ketogenic, Athlete

## Abstract

The purpose of this study was to confirm reduced training metrics previously associated with a ketogenic low-carbohydrate (CHO) high-fat diet (LCHF) and investigate their attenuation with caffeine supplementation. At baseline, *n* 21 elite race-walkers followed a high CHO availability (HCHO) diet and performed a tempo hill session (14 km with a 450 m elevation gain). Athletes were then assigned to either the HCHO or LCHF in a parallel groups design for 3 weeks, where the 14 km tempo hill session was repeated each week. On weeks 2 and 3, in a randomised crossover allocation, all participants received 3 mg/kg caffeine or placebo (gum), 20 min before the session. Race-walking speed, heart rate, ratings of perceived exertion, blood metabolites and Stroop word-colour test metrics were collected. Although LCHF athletes walked faster at baseline compared with HCHO (*P* = 0·049), the HCHO group improved by week 2 (*P* = 0·009) and week 3 (*P* = 0·007), whereas the LCHF group was significantly slower in Week 1 (*P* < 0·001) and Week 2 (*P* = 0·026) compared with baseline. During the 14 km hill session, within-group analysis shows that athletes walked significantly faster (*P* = 0·010) and at a higher percentage of vVO_2_max (*P* = 0·007) when using caffeine compared with a placebo. Between-group differences remained present, with HCHO athletes walking at a higher percentage of vVO_2_max than those adhering to the LCHF diet (*P* = 0·035). No interaction between supplement treatment and dietary group occurred (*P* = 0·640). Caffeine supplementation partially reversed the performance impairment associated with an LCHF diet, but training quality remained lower than the combination of caffeine and high CHO availability.

Adherence to a low-carbohydrate (CHO), high-fat (LCHF) diet has been associated with reduced training quality in competitive endurance athletes^(1)^. Specifically, elite male race walkers who followed a rigorously supervised LCHF diet during a 3-week period of intensified training^(2)^, completed lower training volumes at slower speeds, but equal ratings of perceived exertion (RPE) and higher heart rates (HR), than counterparts who trained with high CHO availability^(1)^. We attributed this to the inferior economy (i.e. increased oxygen cost) of the stoichiometry of fat *v*. carbohydrate oxidation^(3)^, when the muscle becomes highly reliant on fat as an exercise substrate^(4)^. Indeed in comparison to those who trained with CHO support, the LCHF group failed to translate the increased aerobic capacity achieved over the course of the training camp into better performance during a sustained high-intensity race at the completion of the period^(5)^.

Although we explained the race results as an acute effect on exercise capacity in the LCHF group, we acknowledge the presence of some confounding factors in this interpretation. For example, the lack of improvement in race performance could have been underpinned by an attenuated training response, noting that the LCHF group achieved lower training volumes than the CHO-supported groups during the camp. Unfortunately, the design of this study did not include observation of training characteristics prior to the onset of the different diets, preventing us from distinguishing innate characteristics of the group from the effects associated with the LCHF diet on training metrics. However, we reported that the LCHF group achieved a similar relative improvement in training quality (i.e. increase in training speed) over the 3 weeks, as well as a similar relative training load (training volume multiplied by session RPE). Nevertheless, a reduction in absolute training speed, especially during key sessions, could theoretically lead to a reduction in training adaptation and sub-optimal performance gain. Therefore, we were interested to confirm the suspected impairment of higher-intensity workouts associated with the LCHF diet by including baseline measurements prior to the exposure to the diet, as well as to investigate strategies that might ‘rescue’ training capacity.

Caffeine is a well-known performance supplement with robust evidence of benefits across a range of exercise protocols^(6–8)^. Its use during sporting competitions is documented in athlete surveys^(9)^, as well the monitoring of unmetabolised caffeine in urine samples collected during post-event doping control activities^(10)^. Although caffeine is consumed as part of the everyday lifestyle patterns of most adults, the effect of a more targeted use around training sessions to increase training quality and adaptation is underexplored^(11)^. Caffeine reduces perception of effort and increases exercise capacity particularly when used in the fatigued state^(7)^. Furthermore, benefits to endurance exercise are seen with caffeine doses as little as 2–3 mg/kg body mass (BM)^(12)^. Since this dose (e.g. 100–250 g) is within the daily ‘social’/dietary intakes of most adults, it could be repurposed into strategic intake around training sessions as a practical solution to rescue the reduced capacity for high-intensity exercise associated with LCHF adherence. Caffeine-containing gums have become popular among athletes with several commercial products offering the convenience of an effective dose with 1–3 pellets, while being batch testing to reduce the risk of inadvertent intake of substances banned in sport. Furthermore, mastication of caffeinated gum rapidly achieves peak blood caffeine concentrations via buccal cell absorption (i.e. 15 min) compared with liquid or pill forms which are absorbed in the gut (e.g. 45–60 min), providing practical opportunities for targeted enhancement of sports performance (for review, see Barreto *et al.* ^(13)^).

Accordingly, the current investigation aimed: (1) To repeat the previous investigation of LCHF and training quality (e.g. absolute and relative speed) with baseline measurements prior to the separate dietary interventions and (2) to explore how low-dose caffeine affects performance in athletes who follow LCHF diets during a typical high-intensity endurance session. Noting unpublished observations of mental fatigue in our previous studies of the LCHF diet in elite athletes underdoing intensified training^(5,14,15)^, we also examined the impact of caffeine supplementation on cognitive performance.

## Methods

The Supernova 2 project, in which the current study was a sub-investigation, has been previously described^(14)^. Briefly, elite male and female race walkers undertook a research-embedded training camp while resident at the Australian Institute of Sport in January 2017, after providing informed consent to a protocol conforming to the Declaration of Helsinki and approved by the Australian Institute of Sport Ethics Committee (#20161201). We collected data from twenty-one athletes available from the original twenty-six athletes who participated in the larger study^(14)^.

### Participants

Fifteen male and six female race walkers were recruited for this quasi-experimental investigation, fitting the characteristics of Tier 5 (*n* 1), Tier 4 (*n* 17) and Tier 3 (*n* 3) calibre^(16)^. Athletes were allocated to either HCHO (*n* 8), PCHO (*n* 5) or LCHF (*n* 8) in the original investigation according to a modified patient preference protocol sensitive to considerations in elite sport (Table 1). The athletes in the PCHO group were combined with HCHO for the current study, due to identical energy/macronutrient intakes in total and during the 72 hours prior to each hill session. Furthermore, we noted no differences in training metrics between these groups in the previous study^(1)^. The final characteristics of the sub-study groups were HCHO 8 males and 5 females; 25 (4) years, 63·0 (7·6) kg, 57·5 (3·9) mL/kg/min 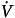 O_2_max and LCHF: 7 males and one female; 29 (3) years, 64·4 (6·6) kg, 61·3 (5·7) ml/kg/min 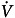 O_2_max. Based on our observations and catering experiences, the group consisted of regular consumers of caffeine in daily diets and social practices and experienced with the use of caffeine as a race aid.

**Table 1.** Rationale and description of methodological design for participant recruitment and treatment allocation

Methodological goals	1. To recruit the highest tier race walkers to the study by ensuring that the individual and their entourage/national sporting organization believed that their involvement would enhance their preparation for the international race calendar 2. To control for individual beliefs about the best nutritional support for training and performance on the motivation for maximal performance efforts 3. To achieve the best possible allocation of treatments (e.g. equal numbers of participants per group with characteristics matched as well as possible) within the confines of the previous goals
Protocol	Overview: Allocation of dietary treatments primarily based on individual preference, with secondary interest to achieve the best obtainable balance of athletic characteristics across groups (e.g. sex, age, personal bests, training history and intended training load during the camp). Specific protocol 1. Provision of potential participants with information about characteristics of each of study treatments (LCHF, periodized CHO availability (PCHO) and sustained high carbohydrate availability (HCHO)^(2)^ and the pros and cons of its use in relation to training and competition. 2. Request for recruited athletes to nominate: preferred treatment; treatments they would be happy to follow and undesirable treatments. 3. First round: allocation of athletes with strong expressions of interest (first choice + opt out choice) noting mean baseline characteristics of each allocation group. 4. Second round: allocation of athletes who expressed interest in trialling more than one diet, balancing group size and participant sex, then other athletic characteristics.
Limitations	Specific criticism that the methodology of assigning participants to their preferred dietary treatment rather than a random allocation is unable to adequately test hypotheses^(17,18)^.
Rationale and benefits	1. Although the randomised controlled trial is a gold standard in study design for estimating the average causal effect of an intervention, in areas of biomedicine in which treatments cannot be blinded, it is acknowledged that strong beliefs by participants can reduce study recruitment, compliance and retention as well as alter study outcomes, particularly for metrics that are subjective or influenced by belief effects^(19)^. 2. The effect of the LCHF diet on sports performance is intensively discussed in lay and social media with polarised viewpoints^(18,20)^. Noting the measurable size of placebo and nocebo effects on this primary outcome of our study^(21)^, our treatment allocation was designed to minimise the nocebo effect and move belief effects in the same (positive) direction. 3. The treatment allocation also accommodated the ethics of requiring elite athletes to undertake a treatment not of their choosing (e.g. perception of coercion) during the preparation and execution of selection for international competition. 4. Issues such as unequal baseline characteristics of groups can be treated with appropriate statistics. Lack of random allocation of treatments requires careful interpretation and can preclude conclusions of causative relationships. However, it is also noted that observations of reduced performance in athletes who have elected to follow the diets in the expectation of benefits should be carefully considered.

LCHF, low-carbohydrate high fat; CHO, carbohydrate; PCHP, periodised carbohydrate availability; HCHO, high carbohydrate availability.

### Experimental design

**Figure 1.**
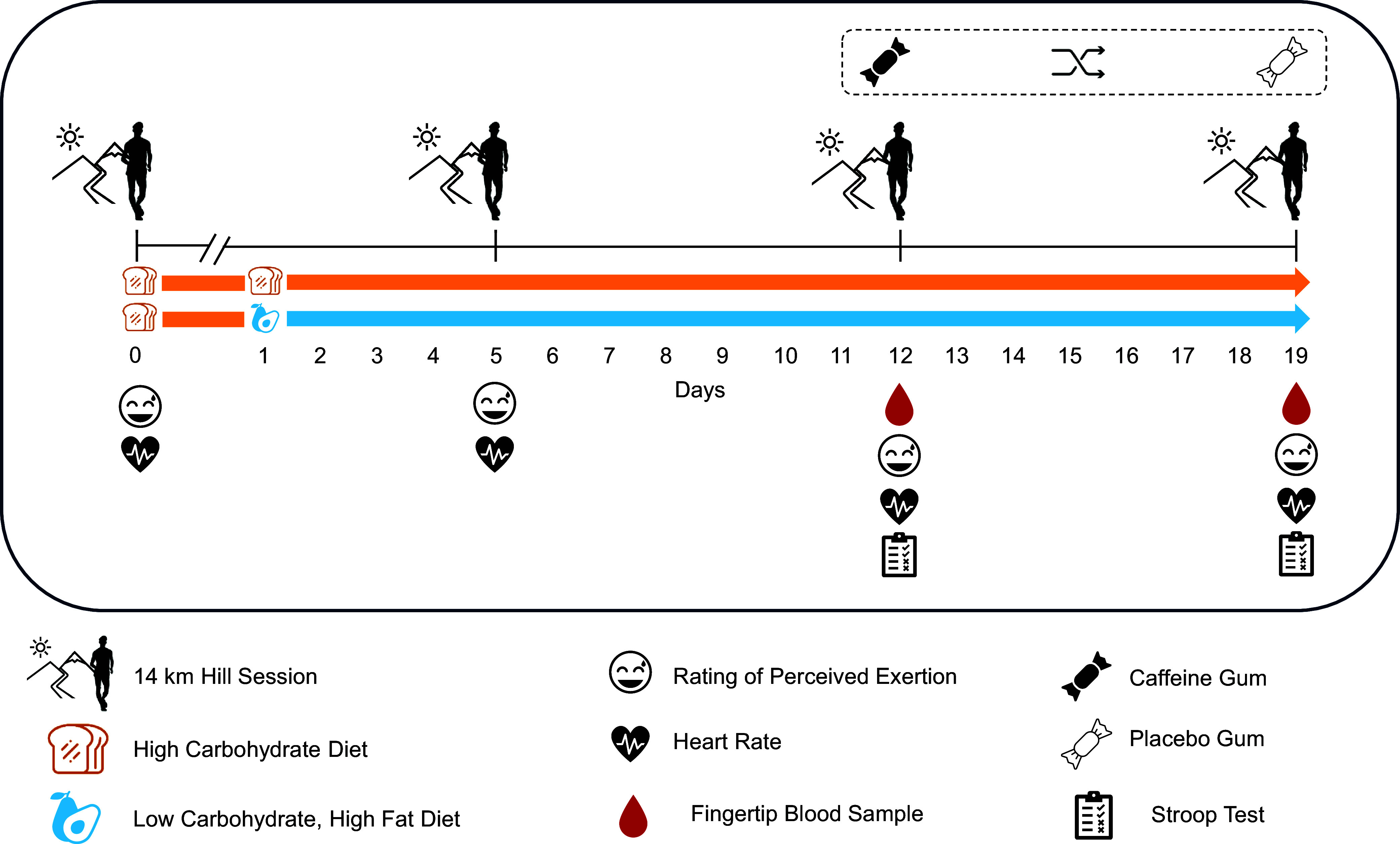
A caffeine *v*. placebo trial assessing 14 km walking performance, cognitive performance and blood metabolites, embedded in the Supernova 2 training camp where athletes were adhering to either a HCHO diet (*n* 13) or LCHF diet (*n* 8). LCHF, low-carbohydrate high fat; CHO, carbohydrate; HCHO, high carbohydrate availability.

At the start of the camp, athletes were asked to complete a baseline 14 km tempo hill training session and a treadmill-based VO_2_max race walking test. Both tests were undertaken with high CHO availability. Using a parallel groups design, athletes were then allocated to follow one of two different dietary approaches to support the training program, either continued high CHO availability or sustained LCHF. Over the next 3 weeks, athletes completed an additional three 14 km tempo hill training sessions, in additional to five compulsory racewalking sessions per week. On top of this, athletes self-selected additional racewalking or cross-training sessions (e.g. running, swimming, cycling and resistance training). In week 2 and 3, all athletes participated a cross-over protocol investigating the effects of pre-session caffeine supplementation on race-walking and cognitive performance. A graphical overview of the larger study design has been represented in Figure 1.

### Dietary intervention

The protocol for designing and supervising the intake of all food and fluids consumed throughout the study has been previously outlined^(2)^, In weeks 1–3, the following dietary interventions were implemented^(14)^:

HCHO (*n* 13): 24-h pre-session: ∼8 g CHO/kg BM (63–65 % energy); 2·0 g protein/kg BM (15 % energy). Pre-session (60–90 min) snack: 1–1·5 g CHO/kg BM. During session: 600 ml sports drink (∼40 g CHO)

LCHF (*n* 8): Energy-matched ketogenic LCHF diet based on principles, menus and recipes supplied by popular diet book^(22)^ and consumed for at least 4 d before week 1. Mean daily intake: ∼0·5 g CHO/kg BM (< 50 g/5 % energy); ∼78 % fat and ∼2·2 g.kg protein (16 % energy). Pre-session: LCHF snack. During session: 600 ml electrolyte drink.

### VO_2_max test protocol

A treadmill-based incremental exercise test was performed under laboratory conditions to determine each athlete’s VO_2_max. Briefly, after 4-submaximal stages, participants completed a ramp (speed and then gradient) test to volitional fatigue. Treadmill speed was increased by 0·5 km/h^–^ every 30 s for a total of 4 min, followed by treadmill gradient increases by 0·5 % thereafter until exhaustion. Velocity at VO_2_max (vVO_2_max) was then calculated using slopes created from treadmill speed, measured VO_2_ and the athletes VO_2_max. This protocol has been explained in more detail elsewhere^(14)^.

### Tempo hill exercise session

In total, four tempo hill sessions were completed during the training camp. The first session (baseline) was completed prior to the allocation of dietary interventions where all athletes had high CHO support. The next session (week 1) was performed after adhering the dietary intervention for 5 d. The final two sessions undertaken in weeks 2 and 3 were also used to study the effects of pre-exercise caffeine supplementation (see Figure 1). All sessions were undertaken on the same outdoor 14 km road circuit in Canberra, Australia. The course had ∼450 m elevation and athletes were instructed to complete the course in the fastest time possible. During each session, heart rate (HR) (Forerunner, Garmin International) and rating of perceived exertion (RPE; 6–20, Borg Scale) were reported at the session end. Time to complete each session was recorded and used to calculate average walking speed, reported as total completion time (min) and speed as a percentage of *v* 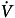 O_2_max.

### Pre-Exercise caffeine supplementation

On weeks 2 and 3, athletes consumed either a gum providing 3 mg/kg caffeine (Stay Awake Military Gum) or placebo gum, 20 min prior to starting the 14 km tempo hill raining session. Doses of caffeine were calculated based on BM and rounded up to the nearest 25 g (e.g. a 60 kg athlete received 200 mg caffeine, a 70 kg athlete received 225 mg caffeine) with the 100 mg caffeine gum pellets being cut into quarters to achieve the desired dose. Modelling of intakes of individual athletes estimates the effective dose was 3–3·4 g/kg, with no expectation of a dose–response within this range.

The allocation of treatments occurred in a randomised cross-over design, such that all athletes completed both the caffeine and the placebo conditions. We collected fingertip capillary blood samples, pre- and post-session, to provide measurements of lactate (Lactate Pro 2, Arkray), ketone bodies (ß-hydroxybutyrate (ßHB)) and glucose (Freestyle Optimum Neo, Abbott Diabetes Care).

### Cognitive performance

Immediately before and after completion of the session of the tempo hill session on week 2 and 3, a 3-min Stroop Word Colour Test (Stroop Test) was administered using electronic tablets (Stroop Test @NovakSportSci). This neuropsychological test measures an individual’s ability to inhibit cognitive interference by measuring speed and accuracy during a task to name colours that are written in matching (congruent) or different colour (incongruent) ink^(23)^. This test was conducted immediately before and after completion of the session with participants being familiarised to this novel task by providing access to the electronic tablets at meals. Based on data generated by the application, the number of questions answered and the number of correct answers and time taken per correct score were recoded for each 3-min testing period.

### Statistical analysis

Data from twenty-one athletes were included in analysis. Data were initially assessed for normal distribution via visual inspection of QQ- and residual plots and Shapiro–Wilk tests for conducted. Data were normally distributed expect for lactate and ketone concentrations, which were log-transformed prior to analysis, and HR and RPE where non-parametric tests were used. Using R-Studio (version 2024.09.1), two sets of linear mixed-effects models were constructed using the lme4 package. Models assessing the impact of the dietary intervention included fixed effects for group (HCHO *v*. LCHF), time (baseline, week 1, week 2 and week 3) as well as the group×time interaction. A random intercept for participant was include to account for repeated measures. The outcome variables were absolute and normalised race walking speed. To assess the impact of pre-exercise caffeine ingestion, similar models were created using fixed effect for treatment (caffeine *v*. placebo), group (HCHO *v*. LCHF), where appropriate time (pre- *v*. post-exercise) and their interactions. A random intercept for participant was included to account for repeated measures. Outcome variables included race walking speed (absolute and normalised), cognitive performance and metabolites. VO_2_max was included as a covariate in performance-related models (absolute and normalised race walking speed) to account for the differences in baseline characteristics and aerobic capacity. *P* values were obtained using Type II Wald F tests with Kenward–Roger degrees of freedom approximation. Where significant fixed or interaction effects were evident, pairwise comparisons were performed using estimated marginal means with Tukey adjustment for multiple comparisons (emmeans package). Significance was set at *P* ≤ 0·05, and Cohen’s d effect sizes were calculated to show within group effects. Data are presented as mean (sd), except for HR and RPE, which is presented a median ± IQR.

## Results

### Dietary intervention

Methods to maximise compliance to the dietary protocols and to measure adherence (via biomarkers such as blood concentration of ketone bodies) over the study duration are provided in the primary publication, as is the macronutrient breakdown of each intervention^(2)^.

#### Race walking performance

**Figure 2.**
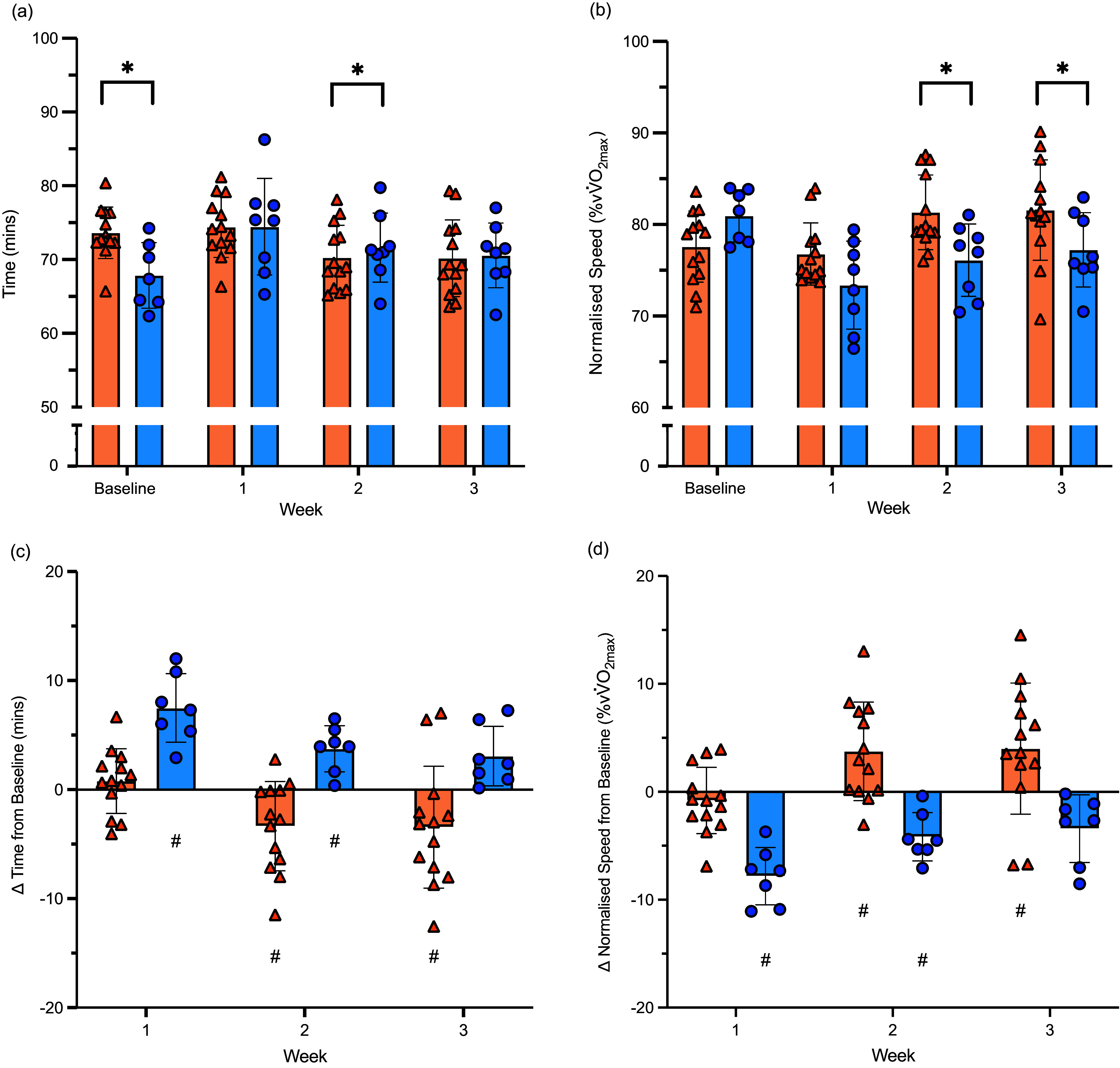
Weekly hill session results for athletes on HCHO (*n* 13; orange) and LCHF (*n* 8; blue) diets. (a) 14 km hill completion time. (b) Hill walking speed normalised to vV˙O_2_max. (c) Change in hill completion time relative to baseline. (d) Change in normalised speed relative to baseline. * indicates a significant difference between groups. ^#^indicates a significant within-group change from Baseline. Data shown are mean (sd) with individual data points. LCHF, low-carbohydrate high fat; HCHO, high carbohydrate availability.

A significant interaction effect (*P* < 0·001) showed that time to complete the tempo hill session was different between dietary groups at baseline (*P* = 0·049), with the LCHF group being faster than the HCHO group (Figure 2(a)). However, during week 1 (*P* = 0·105) and week 3 (*P* = 0·065) of the training camp, tempo hill completion times were no longer different between groups, with the HCHO walking significantly faster than the LCHF during week 2 (*P* = 0·016). When performing a within group comparison (Figure 2(c)), the HCHO group walked significantly faster in week 2 (*P* = 0·009; *d* = 0·83) and week 3 (*P* = 0·007; *d* = 0·77) compared with baseline, whereas the LCHF group was significantly slower in week 1 (*P* < 0·001; *d* = 1·19) and week 2 (*P* = 0·026; *d* = 0·84) compared with baseline. In this model, VO_2_max was a significant covariate (*P* < 0·001), where a higher aerobic capacity was associated with tempo hill completion times.

When walking speed was normalised to *v*.VO_2_max (Figure 2(b)), a significant interaction effect (*P* < 0·001) showed no differences between groups at baseline (*P* = 0·163) or week 1 (*P* = 0·079); however, by week 2 (+5·2 %, *P* = 0·010) and week 3 (+4·4 % *P* = 0·029) athletes on the HCHO diet were able to walk at a higher percentage of *v*.VO_2_max compared with the LCHF group. Within-group analysis showed similar outcomes as time to completion, where the HCHO group walked at a higher percentage of *v*.VO_2_max during week 2 (*P* = 0·005; *d* = 0·93) and week 3 (*P* = 0·003; *d* = 0·84) compared with baseline, whereas the LCHF group walked at a lower percentage of *v*.VO_2_max during week 1 (*P* < 0·001; *d* = 1·90) and week 2 (*P* = 0·011; *d* = 1·40) compared with baseline (Figure 2(d)). In this instance, VO_2_max was not a significant covariate in the model (*P* = 0·708).

Ratings of perceived exertion were typically rated as ‘very hard’ and were similar across all four training sessions in the HCHO (baseline: 18 ± 2, week 1: 17 ± 3, week 2: 17 ± 2, week 3: 17 ± 4; *P* = 0·066) and the LCHF group (baseline: 18 ± 2, week 1: 17 ± 2, week 2: 18 ± 1, week 3: 17 ± 3; *P* = 0·331). Average HR reduced over time in the HCHO (*P* = 0·002) with significant differences evident between baseline (170 ± 5) and week 3 (158 ± 24). No differences were evident over time in LCHF (baseline: 165 ± 20, week 1: 161 ± 15, week 2: 170 ± 10, week 3: 166 ± 13; *P* = 0·630).

### Pre-exercise caffeine supplementation

#### Race walking performance

**Figure 3.**
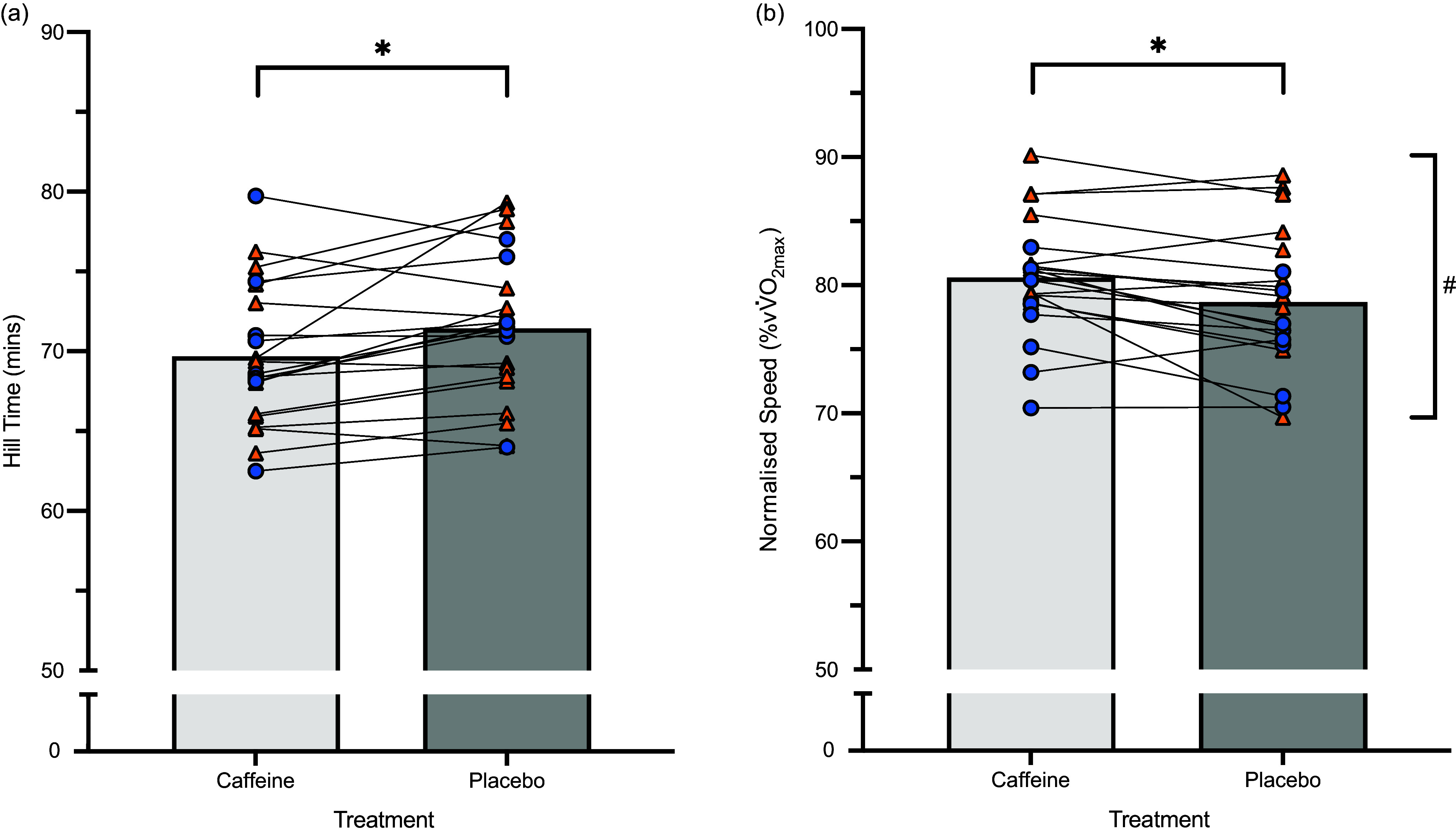
Hill session results for athletes (*n* 21) on HCHO (orange triangles) and LCHF (blue circles) diets, ingesting caffeine (light grey bar) or placebo (dark grey bar). (a) Absolute 14 km hill session completion time (b) 14 km hill walking speed normalized to vV˙O_2_max. *Significant improvement in hill completion time/normalised speed. ^#^Significant difference between HCHO and LCHF diets. Data are means and individual results. LCHF, low-carbohydrate high fat; HCHO, high carbohydrate availability.

A fixed effect of treatment (*P* = 0·010) showed that time to complete the tempo hill session was significant faster with caffeine than placebo (2·4 %; *d* = 0·37), and there was no interaction with the dietary intervention (*P* = 0·640; (Figure 3(a)). In this model, VO_2_max was a significant covariate (*P* < 0·001), where a higher aerobic capacity was associated with faster tempo hill completion times. When speed was normalised to *v*VO_2_max, significant effects for both treatment (*P* = 0·007) and dietary group (*P* = 0·035), but no interaction effect (*P* = 0·693) or relationship with VO_2_max (*P* = 0·998) was evident. Indeed, all athletes walked at a high percentage of their VO_2_max when using caffeine (+2·39 %; *d* = 0·38), while independently, athletes adhering to the HCHO diet walked at a higher *v*VO2max compared with athletes adopting the LCHF diet (Figure 3(b)). Perceived ratings of exertion (caffeine *v*. placebo: 17 ± 1 *v*. 17 ± 2; *P* = 0·637) and heart rate (166 ± 10 *v*. 160 ± 23; *P* = 0·059) did not differ across treatments.

#### Cognitive performance

**Figure 4.**
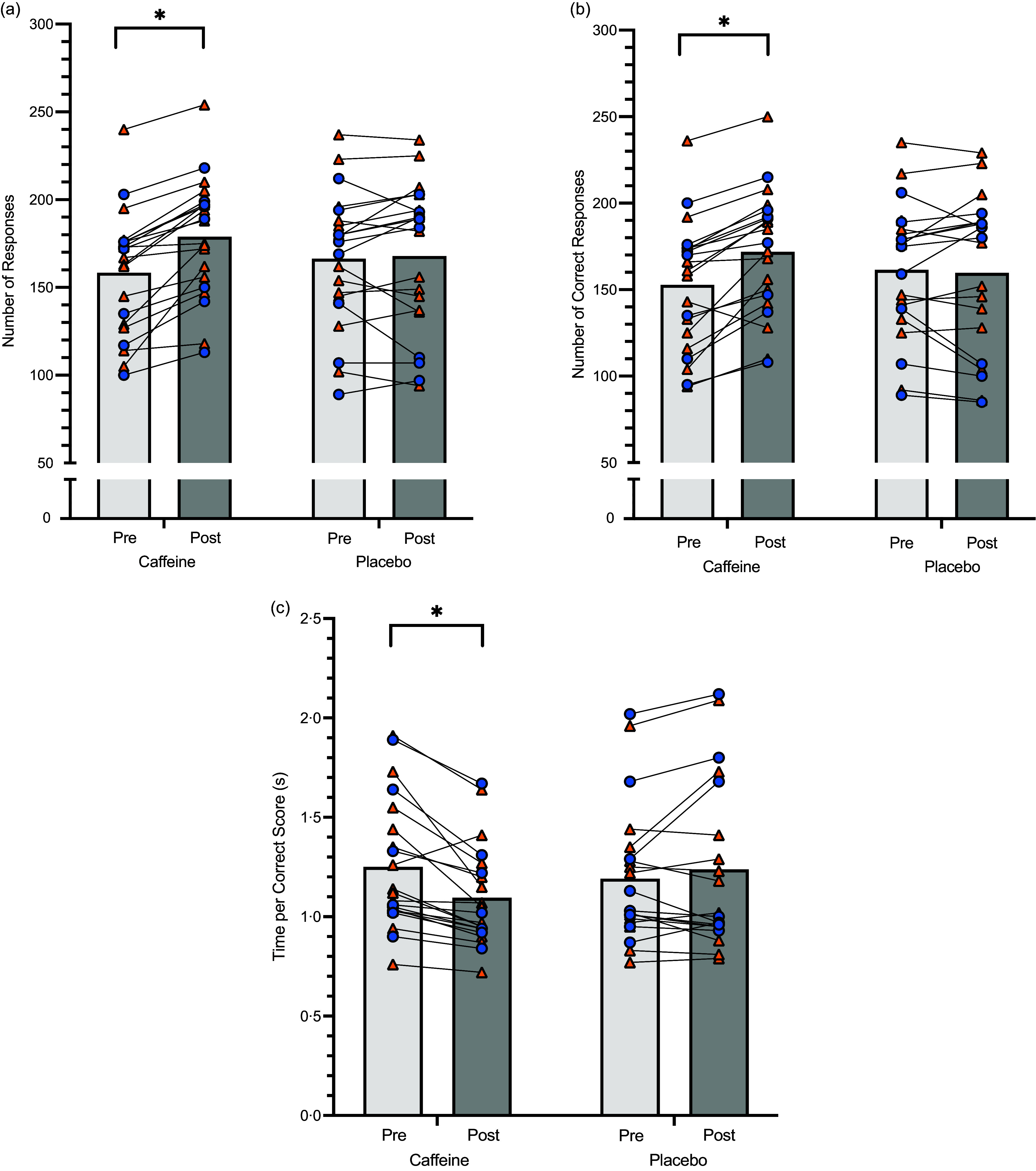
Stroop test results for athletes (HCHO diet – orange triangles, LCHF diet – blue circles) pre- and post-exercise (light and dark grey bars, respectively) having consumed caffeine or placebo (*n* 21). (a) Number of questions answered, (b) number of correct answers, (c) time taken per correct score. *Significant improvement in performance outcome pre- to post-exercise with caffeine ingestion. Data are mean and individual scores. LCHF, low-carbohydrate high fat; HCHO, high carbohydrate availability.

Caffeine ingestion improved outcomes of the Stroop test relative to placebo, irrespective of diet (all diet effects *P* > 0·05). There was an interaction between treatment and time (*P* < 0·001), with athletes answering more test questions post-exercise than pre-exercise, after caffeine (*P* < 0·001), but not placebo (*P* = 0·729) ingestion (Figure 4(a)). Significant interaction effects (*P* < 0·001) showed that athletes also answered more questions *correctly* post-exercise compared with pre-exercise after caffeine (*P* < 0·001), but not placebo (*P* = 0·627) ingestion (Figure 4(b)). Consequently, this resulted in a faster time per correct score post-exercise relative to pre-exercise after caffeine intake (*P* < 0·001), but not placebo ingestion (*P* = 0·211; Figure 4(c)).

#### Metabolites

**Figure 5.**
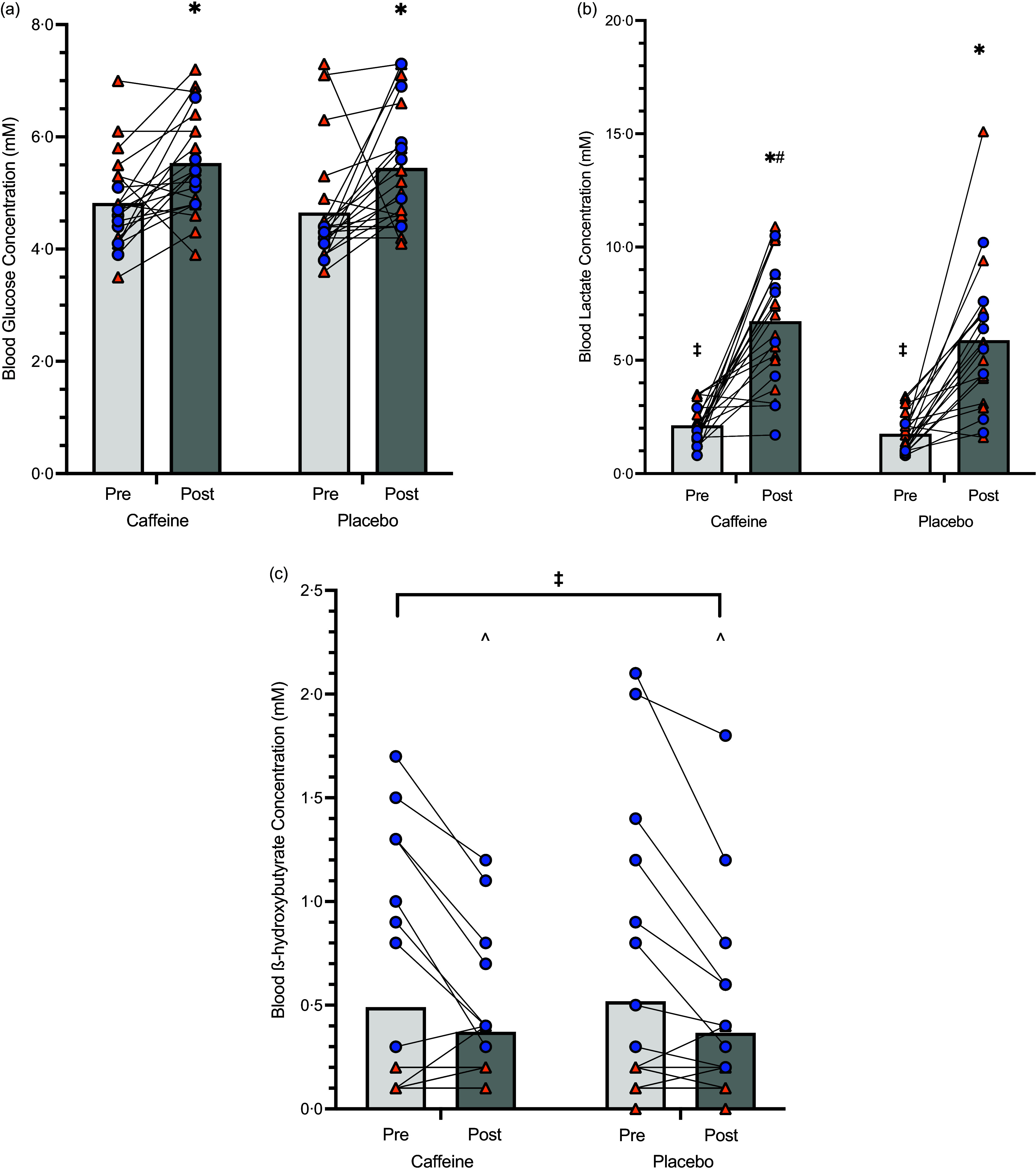
Blood glucose (a), lactate (b) and ketone concentrations (c), pre- and post-14 km hill session when ingesting caffeine or placebo (*n* 21). Light grey bars represent pre-exercise, and dark grey bars represent post-exercise. Orange triangles represent HCHO diet, and blue circles represent LCHF diet. *indicates a significant difference between pre- and post-exercise. ^ indicates a significant difference between pre- and post-exercise in LCHF athletes only. ^†^ indicates a significant difference between the HCHO and LCHF group. # indicates a significant difference between the caffeine and placebo trials. Data shown as mean and individuals’ concentrations. LCHF, low-carbohydrate high fat; HCHO, high carbohydrate availability.

Blood glucose concentrations increased post-exercise (*P* < 0·001); however, no treatment (*P* = 0·510) or diet (*P* = 0·247) effect was evident (Figure 5(a)). A significant treatment effect for lactate concentrations was evident (*P* = 0·039), where concentrations were higher during the caffeine trial compared with placebo (Figure 5(b)). Significant interactions between diet and time (*P* = 0·033) were also found, showing pre-exercise lactate concentrations were lower in the LCHF group compared with HCHO (*P* < 0·001); however, no differences were seen between groups post-exercise (*P* = 0·427). A significant time–diet interaction effect (*P* < 0·001) showed that ketone concentrations significantly decreased from pre-exercise to post-exercise in the LCHF group (*P* < 0·001), whereas concentrations remained similar within the HCHO group (*P* = 0·05; Figure 5(c)). Furthermore, the LCHF had significantly higher concentrations than the HCHO at both pre- and post-exercise (*P* < 0·001).

## Discussion

These results support our previous findings^(1)^, but with a superior methodological design, that adherence to a ketogenic LCHF diet is associated with impaired training capacity during key high-intensity sessions in high performance endurance athletes. Indeed, race walking performance was reduced in elite (Tier 3–5) male and female race walkers who followed a LCHF diet, negating their previous performance advantage over a control group who followed CHO-supported training. This finding did not diminish over 3 weeks of dietary adherence or training. Supplementation with a rapid-acting caffeine source (caffeinated gum) prior to the training session achieved the expected (∼2–3 %) improvement in time to complete the tempo hill session, as well as a post-exercise improvement in a practiced cognitive test. Physical and cognitive benefits associated with caffeine supplementation were similar between groups. Therefore, although the loss of training quality associated with the LCHF diet was partially rescued with caffeine supplementation, it remained reduced in comparison to the combination of caffeine and high CHO availability. The elements of novelty in this study include the focus on training performance, the involvement of elite athletes and the collection of data from real-world training sessions. We also note the involvement of female athletes within the cohort, contributing to the call for their increased participation in sports science investigations^(24)^ although without opportunity to examine any specific sex-related issues in the current study design.

Because other studies of LCHF interventions in endurance athletes have neither included high performance athletes nor systematically quantified training responses, we have been limited in the application of the general literature on keto-adaptation and endurance performance to the current theme. Nevertheless, we draw the reader’s attention to other studies of longer-term (> 3 week) adaptation to LCHF diets which have failed to find impairments of exercise performance/capacity during low-moderate intensity exercise and/or sub-elite participants^(4,25–27)^. With regard to specific interest in elite athletic populations^(5,14,15,28)^, we have found consistent associations with impaired performance of real-world higher-intensity (> 80 % VO_2_max) sporting events in elite race-walkers, using designs that integrate rigorous application of the dietary interventions, tightly standardised protocols for monitoring outcomes and authenticity with measuring athletic performance. Potential mechanisms include a reduction in exercise economy (increase in oxygen cost) that has been consistently measured in these studies as well as those from other laboratories^(4)^ and explained by the stoichiometry of the pathways of fat *v*. CHO oxidation^(3)^. However, in the studies involving longer (∼25 d) exposure to the LCHF diet^(5,14)^, a reduction in training quality, particularly of key higher-intensity workouts within the program, could provide a secondary mechanism. Indeed, in the first study, we reported that participants following the LCHF diet showed impaired training capacity relative to their counterparts following CHO-supported training, with lower training volumes at slower speeds, but higher perceptual (RPE) and physiological (HR) effort for any given speed^(1)^. However, without baseline training data, comparisons could only be made between groups during the dietary interventions rather than being anchored to pre-intervention characteristics, and we acknowledged uneven matching of the groups, with the LCHF group displaying a trend for higher aerobic capacity and personal bests^(5)^.

The current study addressed the former limitation by capturing baseline data for the key training session on which this paper is focussed. The comparison between- and within-trials showed a reduction in speed and training performance in the group undertaking the LCHF diet over the 3 weeks of the intervention (Figure 2). Whereas the LCHF group was faster (∼6 min) and walked at a speed representing a higher (∼3·4 %) percentage of their maximal aerobic capacity at baseline compared with the HCHO group, these differences were negated over the 3 weeks of the LCHF intervention. Indeed, absolute times to complete the tempo hill session for the LCHF group were slower by ∼7 min (week 1) to ∼ 3 min (week 3) compared with a 3·5 min improvement (week 3) in the HCHO group from baseline. Although the LCHF group walked at a speed representing a higher percentage of their aerobic capacity at baseline (∼80 % VO_2_max), their reduced speed on the CHO-restricted diet accounted for an apparently lower percentage of this ceiling and a significantly lower normalised speed (∼5 %) than the HCHO group for weeks 2 and 3. Nevertheless, the metabolic characteristics of the session (e.g. perceived effort and heart rate) remained similar between groups.

We acknowledge that we combined data from two dietary groups in the original study design (PCHO and HCHO) in this sub-analysis. Although these were separate approaches to supporting overall training adaptation and performance in the larger investigation, in both of the studies in which we have compared these approaches^(5,14)^, as well as a separate investigation in elite athletes^(29)^, no additional benefits were achieved by including dietary manipulations to lower muscle glycogen content prior to completing 2–3 sessions within each training week. The authors of the independent study concluded that ‘superimposing periodic CHO restriction to 4 weeks of regular endurance training had no superior effects on performance and muscle adaptations in elite endurance athletes’^(29)^. It appears that the physiological characteristics of elite athletes and/or the glycogen-altering characteristics of the volume/intensity of their training do not respond to periodic implementation of recover-low/train-low dietary strategies in the same manner as athletes of lower tier/lower training volume (for review, see Burke *et al.* ^(30)^). In addition to the lack of differences in global outcomes, PCHO and HCHO athletes in the current study consumed the same foods for the 24-h period prior to the tempo hill session, and the same energy/macronutrient intake for 72 h prior, since the other periodised dietary block occurred at the beginning of the training week. Therefore, we feel justified in treating these athletes as a single cohort for the current analysis.

These findings confirm the concept that the inferred increase in reliance on fat oxidation during sessions, not directly measured during the tempo hill sessions but indicated from other data collected on these participants^(14)^, translates to a lower speed for a given physiological and perceptual effort. Indeed, to provide a completely transparent explanation of our results, we suggest that the tempo hill sessions in the current study were completed by the LCHF group at a similar metabolic (e.g. % absolute aerobic capacity) cost each week. However, because of the reduced economy associated with increased use of fat as an exercise substrate^(4,5,14,15,28)^, the effective speed was reduced to a level associated with a lower percentage of maximal capacity. A similar outcome was suggested in the earlier study from our group and demonstrated in both the training sessions and real-life race performances undertaken by the LCHF group^(1,5)^. However, we noted that training data were reliant on a between-group comparison^(1)^ and in the absence of baseline data from participants, we could not rule out an innate difference between groups to explain the reduced training quality (e.g. reduced training volume, reduced speeds for similar perception of effort during key sessions) associated with the LCHF diet.

Given the physiologically based limitation of higher-intensity exercise with the LCHF diet, we^(15,28)^ and others^(31)^ have investigated strategies that might rescue impaired training or competition performance. The latter involved a case history of a Tier 4 triathlete and long-term follower of the LCHF diet who periodised consumption of CHO immediately before or during higher-intensity aerobic workouts during a 3-week training block before repeating this consumption before/during a series of performance tests^(31)^. Here, the training strategy was undertaken to upregulate intestinal CHO absorption against the background LCHF diet in preparation for the performance battery, and no information was provided on training characteristics other than ‘no subjective difference in training quality or perception of effort was noted’^(31)^. However, within the test battery, CHO ingestion was associated with a worthwhile improvement (2·8 % time, 8 % power) in a 20 km cycling time-trial, without major changes in shorter or longer protocols^(31)^. This confirms the findings of our studies that reliance on fat oxidation is limiting to the performance of exercise in the high-intensity aerobic domain^(5,14)^. The authors of the case study^(31)^ speculated that the improvement of performance associated with CHO intake during exercise could be at least partially attributed to a central nervous system or non-metabolic effect; the so called ‘mouth sensing’ effect^(32)^. A potential disadvantage of this approach is that regular intake of CHO during key training sessions may reduce the period in which the athlete is in the ‘optimal ketosis’ range. The case study did not provide any information on the effect of CHO feeding during the 3-weekly training sessions on blood ketone body concentrations.

Accordingly, a secondary aim of this study was to investigate the effect of caffeine supplementation in rescuing the performance of higher-intensity training sessions without affecting ketosis. As previously described, caffeine is a well-known performance supplement that acts primarily to mask fatigue and perception of effort, with benefits seen at doses of ≥ 3 mg/kg BM^(6,7)^. The outcomes of the current study align with these insights: ∼3 mg/kg BM of caffeine from a fast-acting gum was able to improve the time to complete the tempo hill sessions across the whole group of race walkers by the 2–3 % margin typically associated with caffeine use^(6,7)^. Such an improvement is not only statistically significant but is meaningful to the outcomes of high performance endurance sport in which the coefficient of variation of competition performance is typically less than half of these margins^(33)^. Faster speeds were achieved in the caffeine trial for the same physiological (HR, lactate) and perceptual (RPE) response.

There was no indication of a greater magnitude in the performance improvement with caffeine supplementation in the LCHF cohort compared with the CHO-supported group. Previous studies have suggested that a range of outcomes might have been possible. For example, a previous investigation^(34)^ showed that the same dose (3 mg/kg BM) of caffeine enhanced power output during a high-intensity interval training session in cyclists who had deliberately depleted muscle glycogen stores to train with low CHO availability. However, caffeine supplementation only partially rescued the loss of exercise capacity associated with ‘train low’ strategies, and the best outcome was seen when caffeine supplementation was added to high glycogen availability. Meanwhile, a meta-analysis of twenty-one studies of caffeine supplementation during endurance exercise in which CHO was also consumed (as in our study) reported that the effect size of the caffeine benefit was approximately half (*P* < 0·006) that found in a meta-analysis of thirty-six studies in which caffeine was compared with a non-caloric placebo^(35)^. A potential explanation for the reduced performance benefit of adding caffeine to a scenario of high CHO availability could be an attenuation of the fatigue/perceived effort of exercise with better fuel support, leaving less benefit associated with a fatigue-masking agent. In any case, the results of the current study show that while caffeine supplementation enhanced the performance of the LCHF group, it was unable to bridge the residual reduction in their training capacity compared with their baseline (CHO-supported) performance and the best relative performances of the tempo hill session were associated with HCHO availability and caffeine. This suggests that the impaired performance of the LCHF group in the higher-intensity tempo hill sessions is due to the fundamental issue of reduced energy yield from fat oxidation rather than greater fatigue associated with the early phases of adaptation to the LCHF diet^(1)^.

We also saw an improvement in cognitive performance at the post-exercise testing in the caffeine trial. Results from the Stroop Word Color Test showed an increase in the rate of response (e.g. reaction time), the number of correct responses and, therefore, the time taken to achieve a correct response. Caffeine supplementation is known to enhance alertness, vigilance, attention and reaction time,^(36)^ and some studies of caffeine supplementation in athletes have employed the Stroop Test to measure changes in cognitive performance during or after exercise^(37–40)^. The mixed results from the literature may be attributed to differences in caffeine dose, type of athlete and exercise test and familiarisation with the protocol. The removal of learning effects allowed us to detect clear evidence of cognitive benefits with moderate doses of caffeine at the end of the exercise session (∼1·5 h post-ingestion). There was no evidence of differences in the magnitude of improvement between dietary groups, but future studies should consider using the test at baseline and during the dietary interventions to measure the potential effects of the LCHF on cognitive function. Here, it is noted that there are plentiful anecdotes about the LCHF diet from social and lay media in relation to cognitive function that require systematic examination; these include self-described symptoms of ‘fatigue’, ‘brain fog’ and ‘loss of energy’ from LCHF adoptees in the early phases of adaptation^(41)^ as well as enhanced mental function (‘keto clarity’) associated with long-term adherence^(42)^. Although the latter has been occasionally demonstrated in studies of ketone ester supplementation and sports/exercise performance^(43,44)^, further investigation of the effects of ketosis within the LCHF diet are required.

This study is not without limitations including the small sample size (albeit expected when elite and world class athletes are involved), and the application to high-performance athletes who undertake specific training programmes in which the quality/intensity of sessions is a key to adaptation and performance improvement. The failure to randomise the allocation of participants to treatments is a limitation, as it introduces potential selection bias and limits causal inferences. However, this was an acceptable limitation to the research team to control for the belief effect (see Table 1). Finally, we acknowledge that female athletes merit individual focus in investigations such as this to identify if they have different or special needs in relation to the interventions.

In summary, this study investigated the influence of the ketogenic LCHF diet on training capacity in elite male and female race walkers, by measuring performance during a tempo hill sessions undertaken pre-intervention and at weekly intervals during a supervised 3-week exposure to the diet. Training speed was reduced in the LCHF group over the duration of the dietary intervention, with the removal of their initial performance advantage compared with a group who followed CHO-supported training. Pre-session caffeine supplementation via a rapid-acting caffeinated gum improved the time to complete the tempo hill session by ∼2–3 %, as well as enhancing post-exercise cognitive performance, with no difference in the magnitude of the effect between groups. These findings confirm our previous report of a decrement in training quality associated with the LCHF diet and provide further evidence that this is explained by a fundamental characteristic of the energy-yield associated with fat oxidation rather than transient fatigue. Although athletes who follow the LCHF diet may be able to partially rescue the performance impairment and loss of training quality via caffeine supplementation before specific sessions, their outcomes remain impaired in comparison to the combination of caffeine and high CHO availability.
